# Supplementary material for: Existence of multi-radical and closed-shell semiconducting states in post-graphene organic Dirac materials
Source: Nat Commun. 2017 Dec 5;8:1957. doi: 10.1038/s41467-017-01977-4 (PMC5717056; doi:10.1038/s41467-017-01977-4)
Supplement: Supplementary file 1 — Supplementary Information [file 41467_2017_1977_MOESM1_ESM.pdf]

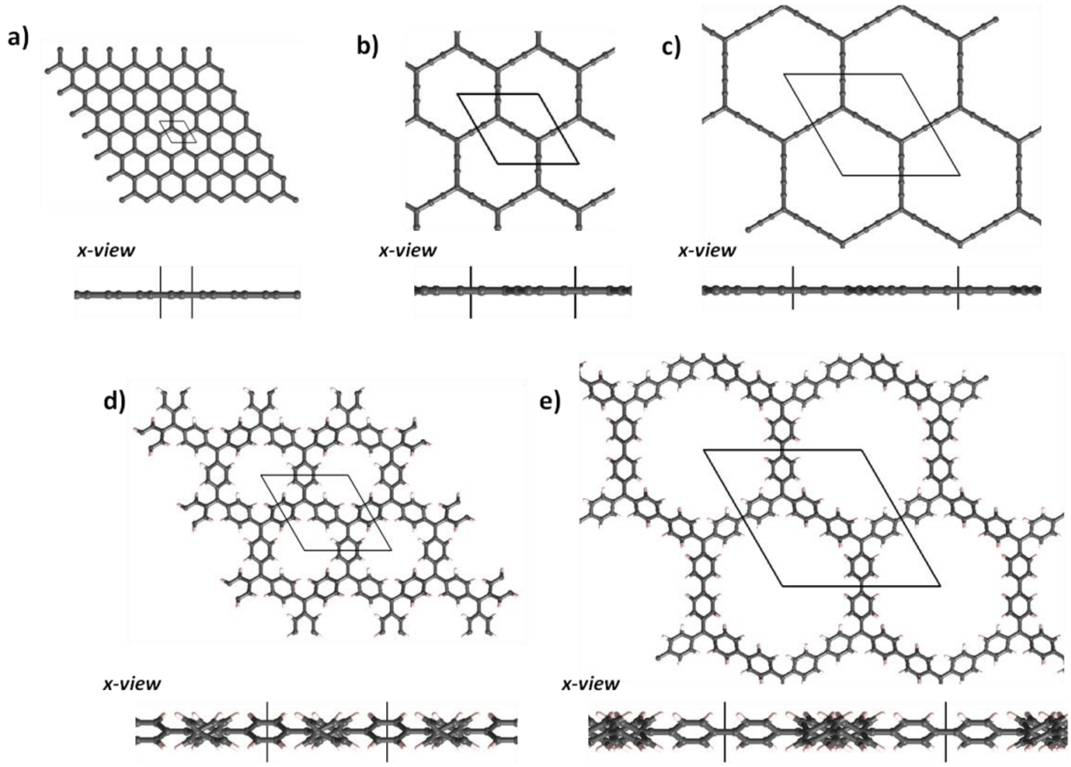

Supplementary Figure 1. **Structures of considered materials.** Fully optimized structures of: a) Graphene, b) GY, c) GDY, d) GP, and e) GDP obtained from unrestricted open-shell DFT based calculations with no initial guess using the PBE functional and the FHI-AIMS code. The utilized unit cells in each case are highlighted from *z*- (upper) and *x*- (lower) views.

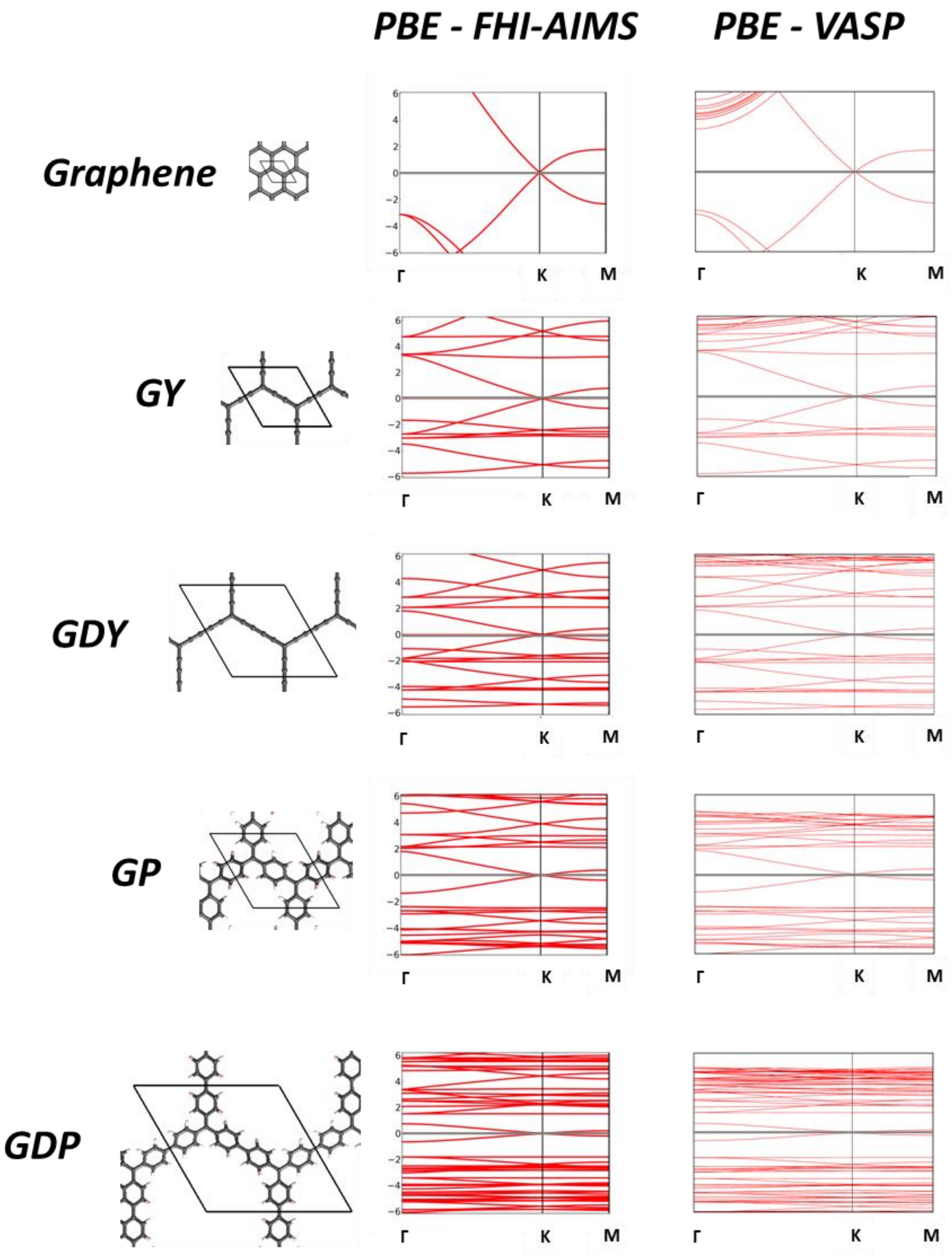

Supplementary Figure 2. Comparison of calculated band structures. Comparison of semimetallic electronic band structures for all considered materials from unrestricted open-shell DFT based optimizations with no initial guess using the PBE functional from FHI-AIMS (left plot) and VASP (right plot) calculations with the corresponding periodic structures (left). The Fermi level is placed at zero energy (horizontal black line), separating the occupied and unocuppied bands. Band structure energy values are given in eV.

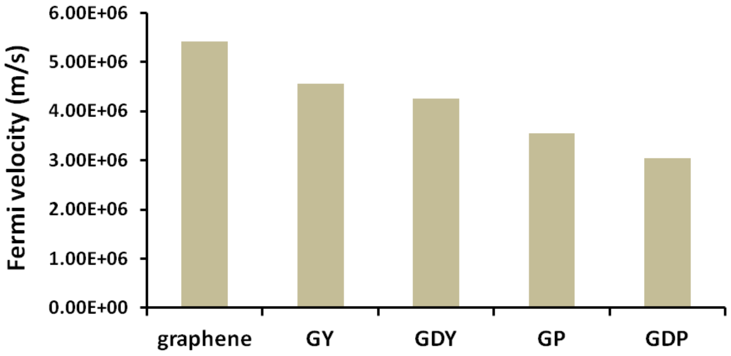

Supplementary Figure 3. Fermi velocities. Calculated Fermi velocites (in metres per second) for each considered material based on band structures from unrestricted open-shell DFT based optimizations with no initial guess using the PBE functional within VASP (see right-hand plots in Suppl. Fig. 2). These values were extracted from the energy *vs*. length slopes in linear segments within the **Γ** → **K** path.

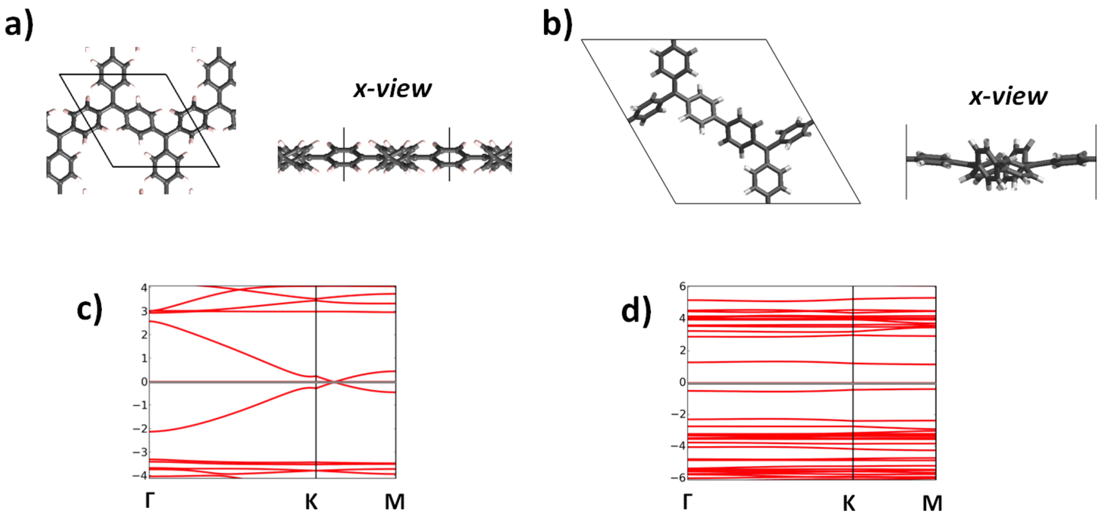

Supplementary Figure 4. Symmetry breaking of GP and GDP. Fully optimized structures of (**a**) GP and (**b**) GDP from unrestricted open-shell DFT based calculations with no initial guess employing the PBE0 hybrid functional and the FHI-AIMS code. The utilized unit cells are highlighted from both the *z*- (left) and *x*- (right) views. In GP one of the three aryl rings within the unit cell becomes very slightly planarised which breaks the perfect hexagonal symmetry. This results in a higher tendency for π-electrons to delocalise in the direction of the more planar ring and a slight shift of the Dirac cone away from the **K** point (**c**). In GDP more significant symmetry-breaking out-of-plane distortions occur (see *x*-view), which results in the opening of a gap at the **K** point (**d**). Band structure energy values are given in eV.

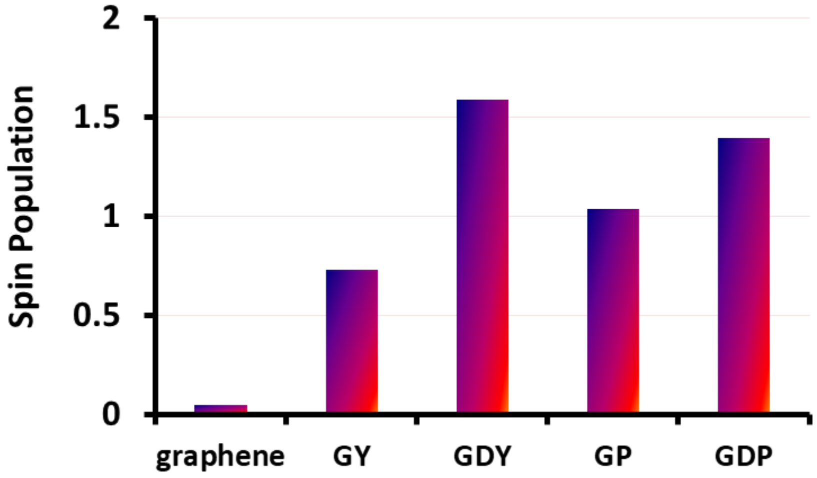

Supplementary Figure 5. AFM spin populations. Spin-unrestricted open-shell single point DFT based calculations employing the PBE0 functional and the VASP code, setting an AFM spin configuration as an initial guess and employing corresponding fully optimized structures from FHI-AIMS calculations. The graph shows the total absolute spin population (i.e. addition of all atom-partitioned spin-up and spin-down electron populations in absolute value) within the unit cell throughout the series of considered materials.

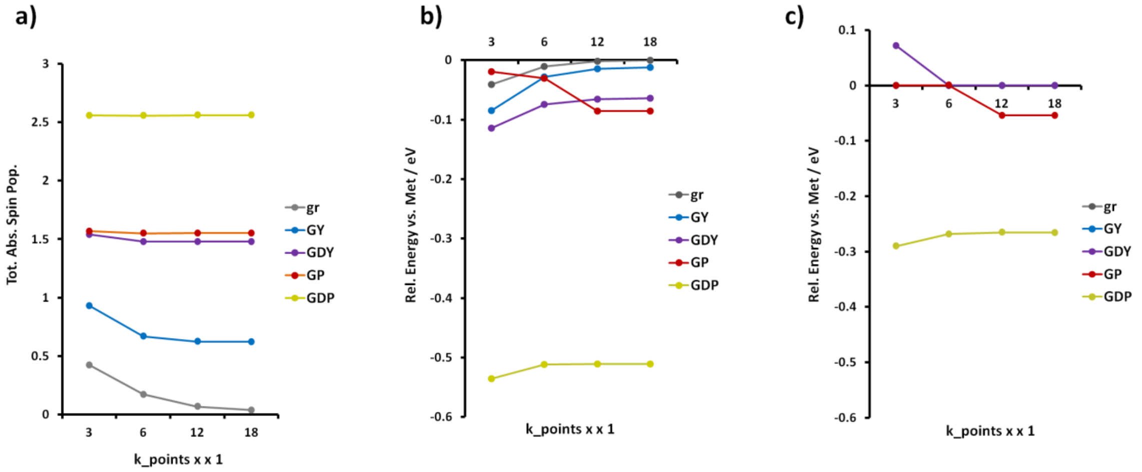

Supplementary Figure 6. k-point dependence of spin populations and relative energies. Summary of results for the AFM open-shell and quinoidal closed-shell solutions from DFT based calculations using the PBE0 functional and the FHI-AIMS code per material upon increasing number of **k**-points utilized in the calculations. (**a**) Total absolute spin population (i.e. addition of all atom-partitioned spin up and spin down populations in absolute value) for the AFM solution within the unit cell throughout the series of considered materials. (**b**) Energy difference (eV) between the AFM solution and the reference semimetallic solution per unit cell. (**c**) Energy difference between the closed-shell solution and the reference semimetallic solution per unit cell. We consider all results to be suitably converged for the 18x18x1 **k**-mesh.

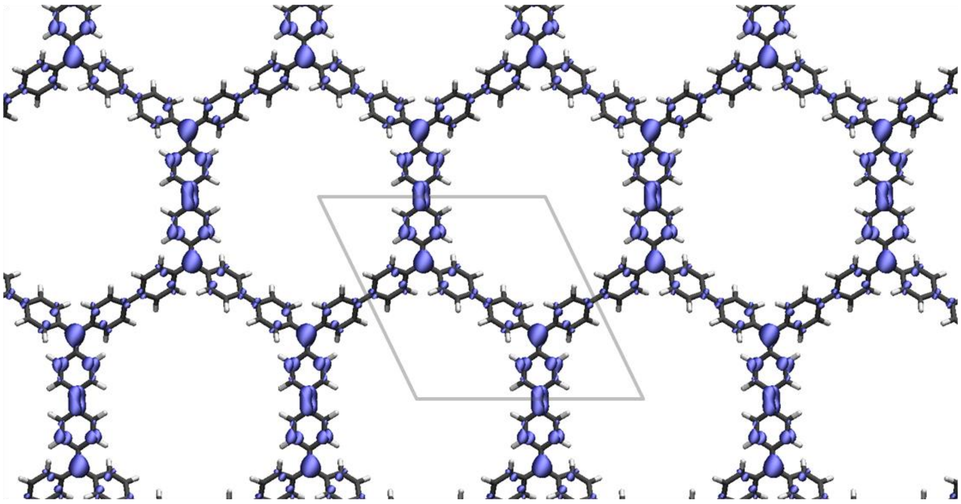

Supplementary Figure 7. Quinoidal state electron density. Atom-projected electron density isosurface of the highest occupied crystal orbital for GDP at the **Γ** point, as obtained from a restricted closed-shell DFT based optimization using the PBE0 functional and the FHI-AIMS code. The isosurface indicates an alternating quinoidal-like pattern of electron pairing between adjacent triply-bonded sp^2 carbon nodes.

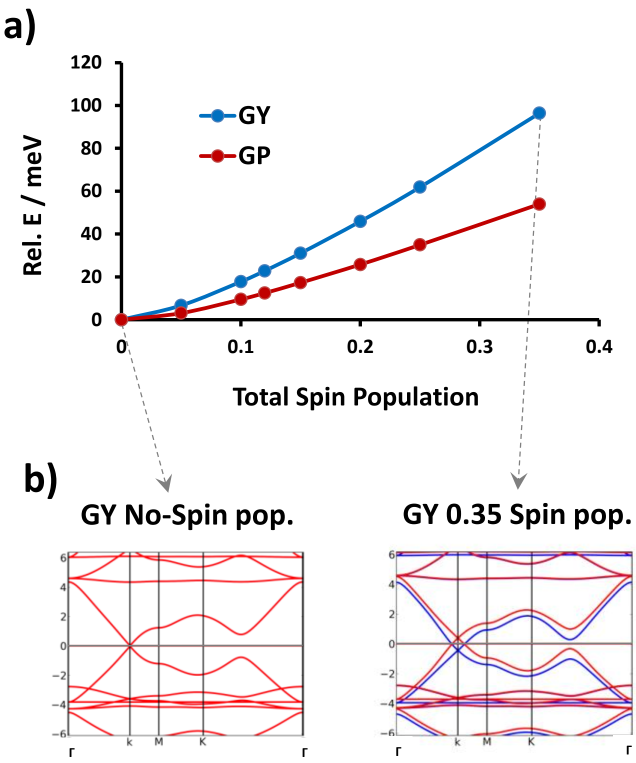

Supplementary Figure 8. Spin polarised solutions for GY and GP. Unrestricted open-shell single point DFT based calculations using the PBE0 functional and the FHI-AIMS code with an increasing spin polarization using the optimised structures obtained for the semimetallic solution at the same level of theory. (**a**) Energy cost (meV) of spin polarizing the semi-metallic solution per sp^2 carbon node for GY (blue) and GP (red) and (**b**) effect on the corresponding band-structures, as shown for GY. As it can be seen, the associated energies are accessible by thermal excitations. Band structure energy values are given in eV.

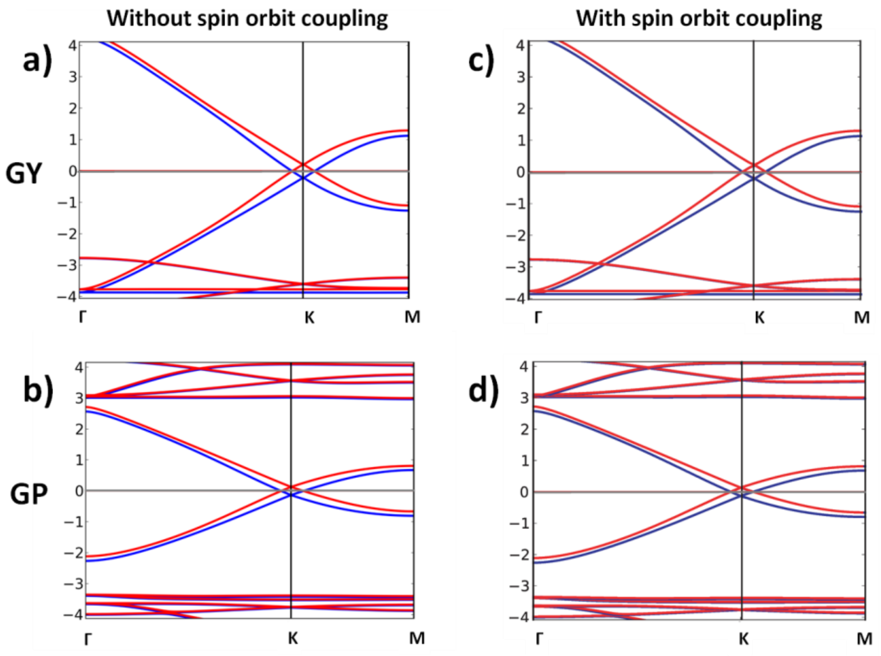

Supplementary Figure 9. Effect of spin-orbit coupling. Unrestricted open-shell single point DFT based calculations forcing a 0.15 spin polarization within the unit cell for the optimized structures for GY and GP in the semimetallic solution using the PBE0 functional and the FHI-AIMS code: (**a**) and (**b**) without spin-orbit coupling, and (**c**) and (**d**) with spin-orbit coupling. Band structure energy values are given in eV.
