# Supplementary material for: Existence of multi-radical and closed-shell semiconducting states in post-graphene organic Dirac materials
Source: Nat Commun. 2017 Dec 5;8:1957. doi: 10.1038/s41467-017-01977-4 (PMC5717056; doi:10.1038/s41467-017-01977-4)
Supplement: Supplementary file 3 — Description of Additional Supplementary Files [file 41467_2017_1977_MOESM3_ESM.pdf]

Description of Additional Supplementary Files

File Name: Supplementary Dataset 1
Description: Atomic coordinates and lattice parameters for optimised structures of all considered materials in the semimetallic solution (calculated using the PBE0 functional and a "light/Tier1" numerical atom-centred orbital basis set as implemented in the FHI-AIMS code)
