# Supplementary material for: Existence of multi-radical and closed-shell semiconducting states in post-graphene organic Dirac materials
Source: Nat Commun. 2017 Dec 5;8:1957. doi: 10.1038/s41467-017-01977-4 (PMC5717056; doi:10.1038/s41467-017-01977-4)
Supplement: Supplementary file 4 — Supplementary Data 1 [file 41467_2017_1977_MOESM4_ESM.pdf]

Graphene

lattice_vector	2.13426496	-1.23221845	0.00000000	
lattice_vector	-0.00000000	2.46449645	0.00000000	
lattice_vector	0.00000000	0.00000000	40.00000000	
atom	-1.42281611	-0.00004457	17.80850200	C
atom	-0.71144885	1.23226302	17.80850200	C

Graphyne

lattice_vector	6.91302568	-0.00000000	0.00000000	
lattice_vector	-3.45270103	5.98507739	0.00000000	
lattice_vector	0.00000000	0.00000000	40.00000000	
atom	4.65948363	2.68773912	19.45338191	C
atom	3.45688398	0.60895349	19.45178344	C
atom	3.45835684	1.99537809	19.45277243	C
atom	2.25809529	2.68909501	19.45355807	C
atom	0.00195557	5.37612818	19.45404627	C
atom	-1.19853217	3.29627677	19.45419743	C
atom	1.20280589	3.29717480	19.45432096	C
atom	0.00193129	3.98972618	19.45432030	C

Graphdiyne

lattice_vector	11.33929472	0.00000000	0.00000000	
lattice_vector	-5.66549818	9.82084601	0.00000000	
lattice_vector	0.00000000	0.00000000	40.00000000	
atom	2.26150421	5.24258715	19.45302200	C
atom	3.41242477	4.57833456	19.45303150	C
atom	5.67039564	0.66445102	19.45303755	C
atom	-2.25729794	5.24271043	19.45304445	C
atom	7.93127765	4.57810591	19.45304499	C
atom	0.00350139	9.15642336	19.45303530	C
atom	0.00211510	6.54736372	19.45303728	C
atom	1.20230178	5.85423863	19.45303485	C
atom	-1.19819520	5.85452652	19.45304562	C
atom	0.00257683	7.93332438	19.45303250	C
atom	4.47162761	3.96667653	19.45302778	C
atom	5.67180758	3.27352379	19.45303500	C
atom	5.67132640	1.88755360	19.45303723	C
atom	6.87214278	3.96633820	19.45303914	C

Graphphenyl

lattice_vector	8.5712763708	-4.9486287200	0.0000000000	
lattice_vector	0.0000000000	9.8972574400	0.0000000000	
lattice_vector	0.0000000000	0.0000000000	40.0000000000	
atom	-2.8570921236	-4.9486287200	-20.0000000000	C
atom	-3.5732997296	-3.7081207577	-20.0000000000	C
atom	-3.0421463316	-2.5433829782	-19.4113646000	C
atom	-2.0876791478	-2.6051042467	-18.9013921200	H
atom	2.8570921236	-4.9486287200	20.0000000000	C
atom	-3.5732997296	3.7081207577	-20.0000000000	C
atom	-1.4246769117	4.9486287200	-20.0000000000	C
atom	3.5732997296	-6.1891366823	20.0000000000	C

atom	3.5732997296	-3.7081207577	20.0000000000	C
atom	1.4246769117	-4.9486287200	20.0000000000	C
atom	3.7237074365	-1.3628845161	-19.4113646000	C
atom	-0.6815611049	3.9062674943	-19.4113646000	C
atom	3.7237074365	1.3628845161	19.4113646000	C
atom	-3.0421463316	2.5433829782	19.4113646000	C
atom	-0.6815611049	-3.9062674943	19.4113646000	C
atom	3.0421463316	2.5433829782	19.4113646000	C
atom	-3.7237074365	1.3628845161	19.4113646000	C
atom	0.6815611049	-3.9062674943	19.4113646000	C
atom	-3.7237074365	-1.3628845161	-19.4113646000	C
atom	3.0421463316	-2.5433829782	-19.4113646000	C
atom	0.6815611049	3.9062674943	-19.4113646000	C
atom	3.2999260310	-0.5054310536	-18.9013921200	H
atom	-1.2122468832	3.1105353003	-18.9013921200	H
atom	3.2999260310	0.5054310536	18.9013921200	H
atom	-2.0876791478	2.6051042467	18.9013921200	H
atom	-1.2122468832	-3.1105353003	18.9013921200	H
atom	2.0876791478	2.6051042467	18.9013921200	H
atom	-3.2999260310	0.5054310536	18.9013921200	H
atom	1.2122468832	-3.1105353003	18.9013921200	H
atom	-3.2999260310	-0.5054310536	-18.9013921200	H
atom	2.0876791478	-2.6051042467	-18.9013921200	H
atom	1.2122468832	3.1105353003	-18.9013921200	H

Graphdiphenyl

lattice_vector	17.51638465	0.00000000	0.00000000	
lattice_vector	-8.76160022	15.17553673	0.00000000	
lattice_vector	0.00000000	0.00000000	20.00000000	
atom	6.93541331	3.39316588	8.83962928	H
atom	6.93067997	0.97107210	8.83529810	H
atom	10.57917230	3.39459423	11.15945044	H
atom	10.58496991	0.97237655	11.16476308	H
atom	8.75652840	5.05559357	9.99824765	C
atom	7.73484278	1.48091686	9.36400600	C
atom	8.75796391	0.73079865	9.99993577	C
atom	7.72728760	2.86190078	9.36872900	C
atom	8.75714718	3.61121996	9.99957497	C
atom	9.78059315	1.48175486	10.63594874	C
atom	9.78741720	2.86276433	10.63073037	C
atom	2.39943994	9.88778182	9.32339726	C
atom	3.59265580	9.18974771	9.31838773	C
atom	1.25149017	9.39437334	9.99823755	C
atom	1.39508919	8.15177753	10.67029985	C
atom	3.74331935	7.95405560	9.99873821	C
atom	2.59605852	7.46690544	10.67592908	C
atom	4.42504786	9.61076305	8.75632195	H
atom	2.32343518	10.82021312	8.76286243	H
atom	2.64582765	6.53438975	11.23630333	H
atom	0.54785467	7.75079669	11.22783372	H
atom	-1.39679438	8.14938226	9.33486070	C
atom	-2.59784327	7.46473980	9.32825327	C

atom	-1.25431144	9.39326925	10.00481111	C
atom	-2.40308151	9.88734525	10.67781323	C
atom	-3.74643980	7.95344016	10.00232103	C
atom	-3.59645738	9.18963991	10.68178173	C
atom	-2.64676460	6.53112377	8.76961743	H
atom	-0.54867174	7.74716425	8.77957155	H
atom	-4.42951495	9.61149541	11.24223398	H
atom	-2.32749531	10.82016787	11.23773525	H
atom	-1.03262621	12.31293681	9.36928643	C
atom	-1.02577160	13.69395878	9.36401159	C
atom	-0.00236145	11.56441410	10.00044342	C
atom	1.02750609	12.31380611	10.63126469	C
atom	-0.00315658	14.44493269	10.00002487	C
atom	1.01994057	13.69480275	10.63597711	C
atom	-0.00174592	10.11994381	10.00179906	C
atom	-1.83014498	14.20333046	8.83517877	H
atom	-1.82440522	11.78113644	8.84057073	H
atom	1.82411014	14.20464871	11.16468303	H
atom	1.81939366	11.78258050	11.16038770	H
atom	6.35547540	5.28766896	10.67662797	C
atom	5.16225773	5.98567620	10.68158869	C
atom	7.50337396	5.78111329	10.00178669	C
atom	7.35983572	7.02367363	9.32970545	C
atom	5.01161943	7.22137429	10.00124030	C
atom	6.15888260	7.70855201	9.32407016	C
atom	4.32985298	5.56465743	11.24362852	H
atom	6.43151392	4.35523657	11.23715815	H
atom	6.10911472	8.64107027	8.76370460	H
atom	8.20710348	7.42462778	8.77220122	H
atom	10.15143906	7.02607401	10.66517320	C
atom	11.35246593	7.71073029	10.67173153	C
atom	10.00901105	5.78221733	9.99521511	C
atom	11.15771410	5.28810950	9.32218573	C
atom	12.50105210	7.22200626	9.99765898	C
atom	12.35108734	5.98579404	9.31821383	C
atom	11.40139401	8.64435534	11.23034846	H
atom	9.30330042	7.42826319	11.22046064	H
atom	13.18414255	5.56393949	8.75776329	H
atom	11.08208275	4.35527785	8.76228626	H
